# Acknowledgement of manuscript reviewers 2015

**DOI:** 10.1186/s40200-016-0222-1

**Published:** 2016-02-22

**Authors:** Bagher Larijani, Farnoush Faridbod, Seyed Masoud Arzaghi, Fatemeh Bandarian, Azadeh Ebrahim-Habibi, Hossein Fakhrzadeh, Shirin Hasani-Ranjbar, Ramin Heshmat, Arash Hossein-Nezhad, Abbas Ali Keshtkar, Patricia Khashayar, Zhila Maghbooli, Ensieh Nasli-Esfahani, Mitra Nourbakhsh, Farshad Sharifi, Akbar Soltani, Farzaneh Zahedi

**Affiliations:** 1 Tehran University of Medical Sciences, Tehran, Iran; 2 Endocrinology and Metabolism Research Institute, TUMS, Tehran, Iran; 3 University of Miami, Coral Gables, USA; 4 Iran University of Medical Sciences, Tehran, Iran

## Abstract

The editors of *Journal of Diabetes & Metabolic Disorders* would like to thank all our reviewers who have contributed to the journal in Volume 14 (2015).

**Ossama Abbas**

Lebanon

**Ebrahim Abbasi**

Iran

**Mohamadsadegh Abedzadeh Zavareh**

Iraq

**Ishag Adam**

Sudan

**Mukesh Mansha Agarwal**

United Arab Emirates

**S Aggarwal**

USA

**Arash Aghajani Nargesi**

Iran

**Lucie Agosta**

USA

**Khodabakhsh Ahmadi**

Iran

**Kiran Ahuja**

Australia

**Ghad Ajabnoor**

Saudi Arabia

**Rola Al Habashneh**

Jordan

**Basma Abdelmoez Ali**

Egypt

**Morteza Alijanpour**

Iran

**Mahtab Alizadeh-Khoei**

Iran

**Samir Al-Minshawy**

Egypt

**Bahareh Amirkalali**

Iran

**Mahsa Amoli**

Iran

**Slobodan Apostolski**

Serbia

**Maryam Ardeshiri**

Iran

**Babak Arjmand**

Iran

**Fariba Asghari**

Iran

**Ambika Ashraf**

USA

**Mr Ashtiani**

Iran

**Evan Atlantis**

Australia

**T Babazono**

Japan

**Salar Bakhtiyari**

Iran

**Farnaz Banakar**

Iran

**Fatemeh Bandarian**

Iran

**Hamid Reza Baradaran**

Iran

**Alaaeldin Bashier**

United Arab Emirates

**Nicola Basso**

Italy

**Sandipan Bhattacharjee**

USA

**Vera Bril**

Canada

**Ae Castro-Sánchez**

Mexico

**Ana Elisa Castro-Sánchez**

Mexico

**Alper Celik**

Turkey

**Saeed Changizi - Ashtiyani**

Iran

**Mohammad Charehsaz**

Turkey

**Maryam Chegini**

Iran

**Qiang Chen**

China

**Kiattawee Choowongkomon**

Thailand

**Concetta Cuscito**

Italy

**Shirin Djalalinia**

Iran

**Shaillay Dogra**

Singapore

**Feixia Dong**

China

**Sandra Dorsainville**

Canada

**Azadeh Ebrahim-Habibi**

Iran

**Mehdi Ebrahimi**

Iran

**Maryam Eidi**

Iran

**Sujindra Elamurugan**

India

**Elhassan Elhassan**

Sudan

**Abdel-Azeem El-Mazary**

Egypt

**Emre Erdogdu**

Turkey

**Rajaraman Eri**

Australia

**Fatemeh Esfahanian**

Iran

**Alireza Esteghamati**

Iran

**Martinsixtus Ezejimofor**

United Kingdom

**Hossein Fakhrzadeh**

Iran

**Susanna Felsenstein**

USA

**Thaís Ferreira**

Brazil

**Narjis Fikri-Benbrahim**

Spain

**Jodok Matthias Fink**

Germany

**John Flack**

USA

**Mirto Foletto**

Italy

**Laercio Franco**

Brazil

**Martha Funnell**

USA

**Jeffrey Gergley**

USA

**Magdalena Gershater**

Sweden

**Richard Gevirtz**

USA

**Mir Farhad Ghaleh Bandi**

Iran

**Asghar Ghasemi**

Iran

**Sambuddha Ghosh**

India

**Masoomeh Gorgian**

Iran

**Elisabeth Govers**

Netherlands

**Danja Groves**

USA

**F Guerrero-Romero**

Mexico

**Pablo Guisado Vasco**

Spain

**Vanessa Ha**

Canada

**Thomas Haak**

Germany

**B Haerian**

Malaysia

**Monir Sadat Haerian**

Iran

**Mahboubeh Hajiabdolbaghi**

Iran

**Mitchell Halperin**

Canada

**Zohreh Hamidi**

Iran

**Kira Harris**

USA

**Shirin Hasani-Ranjbar**

Iran

**Azita Hekmatdoost**

Iran

**Jim Hempe**

USA

**Hossein Hessari**

Iran

**M Horio**

Japan

**Firoozeh Hosseini-Esfahani**

Iran

**Arash Hossein-Nezhad**

Iran

**Silva Hovsepian**

Iran

**Mehrnaz Imani**

Iran

**Urszula Iwaniec**

USA

**Deborah Janowitz**

Germany

**Saad Javed**

United Kingdom

**Muralidharan Jayashree**

India

**Conwoo Emmanuel Jo**

New Zealand

**Pramila Kalra**

India

**Amir Kasaeian**

Iran

**Hatice Kaya**

Turkey

**Alireza Khajavi**

Iran

**A Khajehnasiri**

Iran

**Alireza Khalaj**

Iran

**Mohammadreza Khorramizadeh**

Iran

**Metin Kilinç**

Turkey

**Márcia Klein**

Brazil

**Fariba Koohdani**

Iran

**Melanie Kornides**

USA

**Clement Kufe**

Cameroon

**Gokulakrishnan Kuppan**

India

**D Adam Lauver**

USA

**Elizabeth Lawson**

USA

**Chih-Hsin Lee**

Taiwan

**Ping-Chung Leung**

Hong Kong

**Mahsa M.Amoli**

Iran

**Changsheng Ma**

China

**Zhila Maghbooli**

Iran

**Maryam Mahmoudi**

Iran

**Rayaz Malik**

United Kingdom

**Asieh Mansour**

Iran

**Rudo Mapanga**

South Africa

**Pedro J Marín**

Spain

**Masoud Mardani**

Iran

**Tina Mazaheri**

Iran

**Joan Mcdowell**

United Kingdom

**Antonio Miceli**

Italy

**Alexandros Michopoulos**

Greece

**Mojde Mirarefin**

USA

**Seyed Javad Mirghani**

Iran

**Seyed Mohammad Miryounesi**

Iran

**Amin Mirzaei**

Iran

**Mina Mobasher**

Iran

**Robyn Moffitt**

Australia

**Mohamed Izham Mohamed Ibrahim**

Qatar

**Masumeh Mohammadi**

Iran

**Maral Mokhtari**

Iran

**Seyed Hadi Mousavi**

Iran

**Stephen Mshana**

Tanzania

**Manouchehr Nakhjavani**

Iran

**Mohsen Naseri**

Iran

**Yasser Nassef**

Egypt

**Ali-Akbar Nejatisafa**

Iran

**Nisha Nigil Haroon**

Canada

**Midori Nishiyama**

Japan

**Ahmed Nooh**

Egypt

**Atefeh Noori**

Iran

**Pa Normandin**

USA

**Mitra Nourbakhsh**

Iran

**Azin Nowrouzi**

Iran

**Peter Nthumba**

Kenya

**Youichi Ogawa**

Japan

**Nermin Olgun**

Turkey

**Kobra Omidfar**

Iran

**Christina S Oxlund**

Denmark

**Shadan Pedramrazi**

Iran

**Niloofar Peykari**

Iran

**Mandana Pourian**

Iran

**Flavia Prodam**

Italy

**Mostafa Qorbani**

Iran

**Shadi Rahemzadeh**

Iran

**Shadi Rahimzadeh**

Iran

**P Ranasinghe**

Sri Lanka

**Chythra Rao**

India

**Ali Rasouli**

Iran

**Arezoo Rasti**

Iran

**Luciano Ribeiro**

Brazil

**Lorenz Risch**

Liechtenstein

**C Robertson**

United Kingdom

**Slavko Rogan**

Switzerland

**Akbar Safaei**

Iran

**Mahnaz Sanjari**

Iran

**Katayoun Sargeran**

Iran

**Gunes Senol**

Turkey

**R Serra**

Iran

**Zhaleh Shadman**

Iran

**Gita Shafiee**

Iran

**Mesbah Shams**

Iran

**Alireza Shamsi**

Iran

**Ye Shandong**

China

**Farshad Sharifi**

Iran

**Sara Sheikholeslami**

Iran

**Seyed Vahid Shetab-Boushehri**

Iran

**Shervan Shoaee**

Iran

**Simin Dokht Shoaei**

Iran

**Shahla Shojaei**

Iran

**Meena Shrestha**

Nepal

**Sangeetha Shyam**

Malaysia

**Awadhesh Singh**

India

**Randhir Singh**

India

**Hande Sipahi**

Turkey

**Daphne Smith Marsh**

USA

**Wingyee So**

Hong Kong

**Ghasem Solgi**

Iran

**Akbar Soltani**

Iran

**Ozra Tabatabaei-Malazy**

Iran

**Ehsaneh Taheri**

Iran

**Gholamreza Taheripak**

Iran

**Haleh Tajeddini**

Iran

**Yumie Takeshita**

Japan

**Kumpei Tokuyama**

Japan

**Kamlakar Tripathi**

India

**Surendra Ugale**

India

**J Vaclavik**

Czech Republic

**Mohsen Vahedi**

Iran

**Cynthia Valerio**

Brazil

**Neda Valizadeh**

Iran

**Maryam Vasheghani**

Iran

**Janka Vasková**

Slovakia

**R Venkatesan**

India

**Kerri Viney**

Australia

**Yuedong Wang**

China

**Deng Wei**

USA

**Y Wibowo**

Australia

**Arzu Didem Yalcin**

Turkey

**Mesfin Yimam**

USA

**Kai Hang Yiu**

Hong Kong

**Bedowra Zabeen**

Bangladesh

**Farzaneh Zahedi**

Iran

**Homeira Zardooz**

Iran

**M Zarshenas**

Iran

**Mohammad M. Zarshenas**

Iran

**Roberto Zenteno-Cuevas**

Mexico

